# 한국 성인의 수면 시간과 정신 건강 및 음주 습관의 관계: 2018·2020년 국민건강영양조사 이차자료 분석

**DOI:** 10.7586/jkbns.25.048

**Published:** 2025-11-18

**Authors:** Sujin Lee, Sangeun Jun

**Affiliations:** 1 College of Nursing, Keimyung University, Daegu, Korea; 1 계명대학교 간호대학; 2 College of Nursing, Research Institute of Nursing Science, Keimyung University, Daegu, Korea; 2 계명대학교 간호대학·간호과학연구소

**Keywords:** Sleep, Depression, Stress, psychological, Mental health, Alcohol drinking, 수면, 우울, 스트레스, 정신 건강, 음주

## Abstract

**Purpose:**

This study investigated the associations between sleep duration and depression, stress, perceived health status, mental health counseling experience, and alcohol-related behaviors among Korean adults using data from the Korea National Health and Nutrition Examination Survey (KNHANES).

**Methods:**

A total of 1,656 adults aged 19-64 years with complete responses for relevant variables in the 2018 and 2020 KNHANES were included in the analysis. Sleep duration was categorized according to the American Academy of Sleep Medicine guidelines. Depression was defined as a Patient Health Questionnaire-9 score ≥ 10. Other variables were assessed using structured survey responses and defined following previous studies. The chi-square test and multivariate logistic regression analyses were performed.

**Results:**

Significant differences in depression, stress, and mental health counseling experience were observed across sleep duration groups (short [≤ 6 hours], adequate [7~9 hours], long [≥ 10 hours]). However, alcohol consumption, binge drinking, and perceived health status did not differ significantly by sleep duration. Compared with those reporting adequate sleep, participants with short sleep were more likely to experience stress (odds ratio [OR] = 1.65, 95% confidence interval [CI] = 1.29~2.12), depression (OR = 5.62, 95% CI = 3.00~10.52), and mental health counseling (OR = 3.57, 95% CI = 1.82~7.01). Additionally, those with long sleep duration were more likely to report depression (OR = 5.40, 95% CI = 1.77~16.47).

**Conclusion:**

Inappropriate amounts of sleep, both short and long, was significantly associated with higher risks of depression, stress, and mental health service utilization, whereas no association was found for alcohol-related behaviors. These findings suggest the need for population-level strategies to promote appropriate sleep duration and to improve adult mental health outcomes.

## 서론

### 1. 연구의 필요성

수면은 신체적, 정신적 피로 및 활력을 회복시키고 인체의 항상성 유지 및 정상적인 에너지를 보존에 필수적이며[1], 스트레스와 불안을 경감시키고, 개인의 건강 및 삶의 질에 중요한 영향을 미친다[2]. 적정 수면 시간이란 잠을 자고 일어났을 때, 피곤하지 않으며 낮 동안 졸리지 않고 생활할 수 있는 수면 시간을 말한다. 적절한 수면은 개인의 건강 및 삶의 질에 긍정적인 영향을 미치지만, 부족한 수면은 스트레스, 피로 누적 및 우울증과 불안장애와 같은 정신건강과도 관련되어 신체적 정신적으로 부정적인 영향을 미치게 된다[3].

미국수면재단(National Sleep Foundation)의 권고에 따르면 성인의 적정 수면 시간은 7~9시간이다[4]. 하지만 산업 발달과 스마트기기를 포함한 기술의 혁신은 인간의 삶을 편리하게 만드는 동시에 수면 시간의 감소와 수면 패턴에 부정적인 영향을 초래하고 있다[5]. 실제로 2024년 통계청 조사결과에 따르면, 우리나라 20대 이상의 모든 연령층에서 미디어 이용 시간이 5년 전보다 증가하였으며[6], 국민건강영양조사 결과, 성인의 40.5%가 주중 수면 시간이 7시간 미만으로 수면 부족군에 해당하였다[7]. 또한, Organization for Economic Cooperation and Development (OECD) 통계에 따르면, 한국인의 평균 수면 시간은 조사 대상 국가 중 가장 낮거나 두 번째로 낮아 최하위권에 속한다[8]. 이러한 수면 부족은 개인의 건강뿐 아니라 사회적 생산성 저하, 산업재해와 교통사고 위험의 증가 등 다양한 문제를 유발하여 국가적 경제 부담과 사회적 손실을 초래하는 중요한 보건 문제로 보고되고 있다[9]

국내외 연구에 따르면 수면 부족은 고혈압, 심근경색, 뇌졸중 등의 심혈관계 질환과 인슐린 저항성 증가 등 대사질환의 위험을 높이는 것으로 나타났다[10]. 또한, 수면 부족은 면역 기능 저하 및 암 발생 위험과도 관련되며, 특히 교대근무로 인해 수면-각성 리듬의 교란은 유방암, 전립선암, 대장암 등의 위험이 유의하게 증가하는 것으로 보고되었다[11,12]. 더욱이 수면 부족은 조기 사망률 증가에도 영향을 미치며, 7시간 미만의 수면은 장기적으로 전체 사망률을 증가시키는 요인임이 확인되었다[13].

수면 부족은 정신건강에도 심각한 영향을 미친다. 짧은 수면 시간은 코르티솔 분비를 증가시켜 스트레스 반응을 촉진하고[14,15], 인지 기능 저하, 우울 및 자살 사고 위험을 높인다. 특히 수면 장애는 이러한 정신건강 문제의 발생과 악화를 예측하는 주요 지표로 보고되고 있다[3,16,17]. 최근에는 수면 부족과 음주 습관 간의 양방향적 연관성에 주목한 연구도 증가하고 있다. 불면증 환자는 종종 수면 유도 수단으로 음주를 선택하지만[18,19], 음주는 오히려 수면의 질을 저해하여 수면 부족을 악화시키는 악순환을 초래한다. 따라서, 수면과 음주 간 상호작용에 대한 통합적 분석이 필요하다[18-20].

그러나 기존 연구들은 특정 연령층(청소년, 대학생, 노인 등)에 집중되거나 표본 수가 제한되어 있어 결과의 일반화에 한계가 있으며[21,22], 수면 시간이 정신건강 및 음주 습관에 미치는 영향을 동시에 분석한 대규모 인구 기반 연구는 국내에서 매우 드문 실정이다[23]. 또한 대부분의 연구가 참여자의 주관적 보고에만 의존하여 실제 상담이나 치료 이력과 같은 행동 기반 지표를 충분히 반영하지 못한 한계가 있다.

이에 본 연구는 국민건강영양조사의 국가 단위 데이터를 기반으로 수면 시간과 정신건강(우울, 스트레스 인지, 주관적 건강 인식, 정신 문제 상담 경험) 및 음주 습관(음주 빈도, 폭음 유무)의 관계를 분석하고자 한다. 이를 통해 수면과 관련된 정신건강 및 음주 문제의 예방과 개입 전략 마련을 위한 실증적 근거를 제공하고, 한국 성인의 건강증진을 위한 정책 수립에 기초자료를 제공하고자 한다.

### 2. 연구의 목적

본 연구는 국민건강영양조사의 7기(2018년)와 8기(2020년) 원시자료를 활용하여, 성인의 수면 시간, 정신건강 및 음주 습관의 실태를 파악하고, 수면 시간이 정신건강(우울, 스트레스 인지, 주관적 건강 인식, 정신 문제 상담 경험)과 음주습관(음주 빈도, 폭음 유무)에 미치는 영향을 규명하고자 한다. 이를 통해 수면과 관련된 정신건강 및 음주 문제의 예방 및 관리 전략을 수립하기 위한 기초자료를 제공하고자 한다.

## 연구 방법

### 1. 연구 설계

본 연구는 질병관리청이 실시하는 국민건강영양조사 7기(2018년)와 8기(2020년) 자료를 활용하여, 수면이 음주 습관과 정신건강에 미치는 영향을 분석한 이차 자료 분석 연구이다.

### 2. 연구 대상

**Figure 1.**
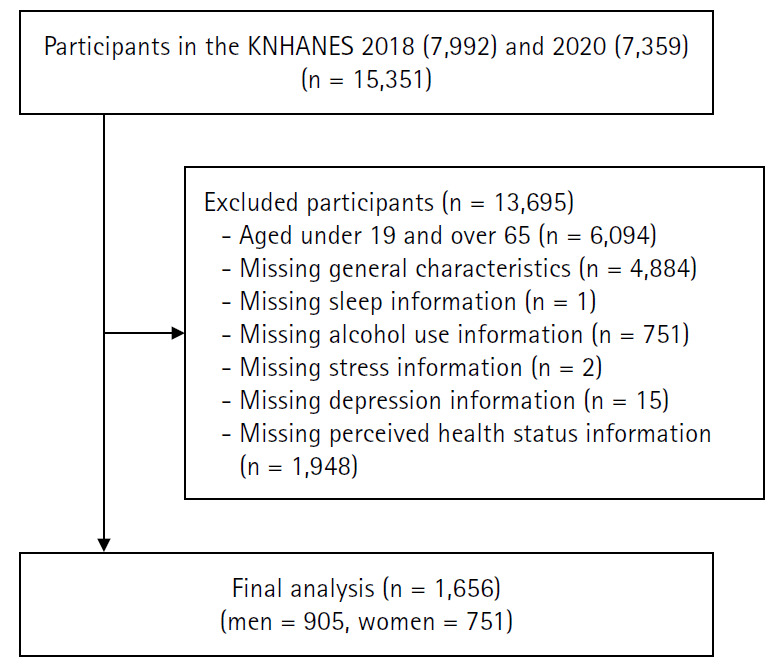
Flow-chart for study participants selection. KNHANES = Korea National Health and Nutrition Examination Survey.

본 연구에서는 7기(2018년) 대상자 7,992명과 8기(2020년) 대상자 7,359명을 통합한 전체 대상자 15,351명 중, 만 19세 이상 만 65세 이하인 9,257명을 대상으로 하였다. 그중에서 연구 변수(수면 시간, 우울, 스트레스, 주관적 건강인지, 정신 문제 상담 경험, 음주 빈도, 폭음 유무)에 결측치가 있는 7,601명을 제외한 1,656명의 데이터를 최종 분석에 사용하였다(Figure 1).

### 3. 연구 도구

#### 1) 일반적 특성

성별, 나이, 교육 수준, 소득 수준, 결혼 여부, 정규직 여부를 포함하였다. 연령은 ‘19~29세’, ‘30~39세’, ‘40~49세’, ‘50~59세’, ‘60~64세’로, 교육 수준은 ‘초졸 이하’, ‘중졸’, ‘고졸’, ‘대졸 이상’으로 구분하였으며, 소득 수준은 소득 사분위 기준금액을 기준으로 ‘하’, ‘중하’, ‘중상’, ’상’으로 구분하였다. 정규직 여부는 ‘정규직’과 ’비정규직’으로 구분하였으며, 결혼 여부는 ‘기혼’, ’미혼’으로 구분하였다.

#### 2) 수면 시간

수면 시간은 주중(또는 일하는 날) 하루 평균 수면 시간을 포함하였다. 본 연구는 선행연구[8]을 바탕으로 수면 시간을 ‘6시간 이하’, ‘7~9시간’, ‘10시간 이상’으로 분류하고, ‘7~9시간’을 적정 수면으로 정의하였다.

#### 3) 음주 습관

음주 습관은 음주 빈도와 폭음 유무로 평가되었다. 음주 빈도는 최근 1년간 음주 빈도로 ‘최근 1년간 전혀 마시지 않았다’, ‘월 1회 미만’, ‘월 1회 정도’, ‘월 2~4회’로 응답한 경우 ‘적정 음주’, ‘주 2~3회 정도’, ‘주 4회 이상’으로 답한 경우 ‘고위험 음주’로 구분하였다[19]. 폭음 유무는 한번에 마시는 음주량을 기준으로 ‘1~2장’, ‘3~4잔’, ‘5~6잔’으로 답한 경우 ‘비폭음’, ‘7~9잔’, ‘10잔 이상’으로 답한 경우 ‘폭음’으로 구분하였다[24].

#### 4) 정신건강

정신건강은 스트레스 인지 정도, 우울, 주관적 건강인지, 정신 문제 상담 여부가 포함되었다. 스트레스 인지 정도는 “평소 일상생활 중에 스트레스를 많이 느끼는 편인가?”의 질문에 ‘대단히 많이 느낀다’, ‘많이 느끼는 편이다’로 응답한 경우는 ‘스트레스 많음’, ‘조금 느끼는 편이다’, ‘거의 느끼지 않는다’로 응답한 경우 ‘스트레스 적음’으로 구분하였다. 우울은 Patient Health Questionnaire-9 (PHQ-9)의 9개 항목에 대하여 최근 2주 동안 얼마나 해당 문제를 겪었는지에 대한 설문으로, ‘전혀 아니다’, ‘여러 날 동안’, ‘1주일 이상’, ‘거의 매일’로, 4점 리커트 척도로 측정되었다. 점수의 범위는 0~27점으로 점수가 높을수록 우울감이 높음을 의미하며, 선행연구를 바탕으로 0~9점을 ‘저우울군‘, 10~27점을 ‘고우울군’으로 구분하였다[25]. 주관적 건강인지는 주관적인 건강 상태에 대한 설문으로, ‘매우 좋음’ 또는 ‘좋음’으로 답한 경우 ‘좋음’, ‘나쁨’ 또는 ‘매우 나쁨’으로 답한 경우는 ‘나쁨’으로 구분하였으며, ‘건강이 좋다, 나쁘다’로 구분되지 않는 ‘보통’은 분석에서 제외하였다[26]. 정신 문제 상담은 1년간 정신 문제로 인해 상담받은 경험의 유무에 대한 설문으로 ‘있음’, ‘없음’으로 구분하였다.

### 4. 자료 분석

본 연구의 분석은 국민건강영양조사 7기(2018년)와 8기(2020년)의 복합표본 설계를 반영하여, 층화, 집락, 표본가중치를 적용한 후 SPSS version 23.0 (IBM Corp., Armonk, NY, USA)을 이용하였다. 연구 대상자의 일반적 특성 및 주요 변수(수면 시간, 정신건강, 음주습관)의 분포는 가중치를 적용한 기술통계로 산출하였다. 수면 시간 그룹(≤ 6시간, 7~9시간, ≥ 10시간) 간 정신건강(우울, 스트레스 인지, 주관적 건강 인식, 정신 문제 상담 경험) 및 음주 습관(음주 빈도, 폭음 여부)의 차이는 복합표본 카이제곱 검정을 통해 비교하였다. 또한 수면 시간이 정신건강 및 음주 습관에 미치는 영향을 확인하기 위하여 복합표본 로지스틱 회귀분석(complex sample logistic regression analysis)을 실시하였다. 분석 결과는 교차비(adjusted odds ratio)와 95% 신뢰구간(confidence interval, CI)으로 제시하였으며, 모든 통계적 유의수준은 양측검정 기준 *p* < .050로 설정하였다.

### 5. 윤리적 고려

본 연구는 계명대학교 기관생명윤리위원회(IRB)의 심의면제를 승인(40525-202502-HR-108-01)을 받아 시행되었다.

## 연구 결과

### 1. 연구 대상자의 일반적 특성과 수면 시간, 정신건강 및 음주 습관 특성

연구 대상자는 남자 60.2% (905명), 여자 39.8% (751명)였으며, 수면 시간은 적정 수준으로 정의한 7~9시간이 57.1% (966명)으로 가장 많았고, 6시간 이하가 41.5% (669)이었다. 음주 습관은 고위험음주가 30.6% (496명)였으며, 한 번에 마시는 음주량을 기준으로 폭음이 43.3% (682명)이었다. 스트레스는 많음으로 응답한 대상자가 29.8% (470명)이었고, 우울감이 높다고 응답한 대상자는 4.2% (65명)이었다. 주관적 건강인지는 나쁨이 26.3% (430명)이었으며, 최근 1년간 정신 문제 상담 여부는 ‘예’가 2.8% (48명)이었다(Table 1).

### 2. 수면 시간 그룹에 따른 정신건강 및 음주 습관 특성 비교

정신건강 특성에서 수면 시간 그룹 간 유의한 차이가 나타났다. 스트레스를 ‘많이 느낀다’라고 답한 비율은 수면 시간 6시간 이하 그룹에서 35.4% (226명), 7~9시간 그룹에서 25.9% (239명), 10시간 이상 그룹에서 19.4% (5명)로, 수면 시간이 짧을수록 스트레스 인지율이 높았다(χ² = 18.28, *p* < .001). 우울 고위험군의 비율은 수면 시간 6시간 이하 그룹에서 7.6% (44명), 7~9시간 그룹에서 1.6% (19명), 10시간 이상 그룹에서 9.6% (2명)로, 통계적으로 유의한 차이가 있었다(χ² = 37.20, *p* < .001).

최근 1년간 정신 문제 상담을 받은 경우는, 수면 시간 6시간 이하 그룹이 4.7% (31명), 7~9시간 그룹이 1.5% (16명), 10시간 이상인 그룹은 2.8% (1명)이었으며, 통계적으로 유의한 차이가 있었다(χ² = 15.14, *p* < .001). 한편 음주 습관, 음주 시 폭음 유무, 주관적 건강인지는 수면 시간 그룹 간에 통계적으로 유의한 차이가 없었다(Table 2).

### 3. 수면 시간이 음주 습관에 미치는 영향

수면 시간이 음주 습관에 미치는 영향을 분석 결과 수면 시간은 음주 빈도(≤ 6 hours, *p* = .914, ≥ 10 hours, *p* = .837)와 폭음 여부(≤ 6 hours, *p* = .635, ≥ 10 hours, *p* = .572)에 통계적으로 유의한 영향을 미치지 않았다(Table 3).

### 4. 수면 시간이 정신건강에 미치는 영향

복합표본 로지스틱 회귀분석 결과, 수면 시간은 스트레스, 우울, 정신 문제 상담 경험과 유의한 관련성을 보였다(Table 4). 스트레스의 경우, 6시간 이하 수면군은 7~9시간 수면군에 비해 스트레스 발생 위험이 1.65배(95% CI: 1.29~2.12, *p* < .001) 높았다. 우울의 경우, 6시간 이하 수면군은 7~9시간 수면군에 비해 우울 발생 위험이 5.62배(95% CI: 3.00~10.52, *p* < .001) 높았으며, 10시간 이상 수면군도 7~9시간 수면군에 비해 우울 발생 위험이 5.40배(95% CI: 1.77~16.47, *p* = .003) 높았다. 최근 1년간 정신 문제 상담 경험은, 6시간 이하 수면군에서 7~9시간 수면군에 비해 3.57배(95% CI: 1.82~7.01, *p* < .001) 더 높았다. 그러나, 수면 시간은 주관적 건강인지에는 유의한 영향을 미치지 않았다

## 논의

본 연구는 국민건강영양조사 자료를 활용하여 우리나라 만 19~64세 대상자의 수면 시간을 확인하고, 수면 시간이 우울, 스트레스, 주관적 건강인지, 정신 문제 상담 경험, 음주 빈도 그리고 폭음 유무에 미치는 영향을 파악하기 위하여 수행되었다.

본 연구에서는 미국수면재단(National Sleep Foundation)에서 지정한 적정 수면 시간인 7~9시간을 기준으로 우리나라 성인의 수면 시간을 분석하였으며, 전체 참여자 중 41.5%가 수면 부족 그룹(6시간 이하)에 해당하였다. 이는 국민건강영양조사 7기(2018년) 자료를 분석한 선행연구에서 보고된 성인의 수면 부족 그룹의 비율이 27.4%로 나타난 것보다 높은 수준으로[27] 최근 국내 성인의 수면 시간이 감소하고 있음을 시사한다. 또한 이는 미국 성인의 수면 부족 비율인 33.2%보다도 현저히 높은 수준이며[28], 일본 후생노동성이 2019년 국민건강영양조사에서 보고한 성인의 수면 부족(6시간 미만 수면자의 비율) 비율(남:37.5%, 여:36.2%)보다도 높은 수준이다[29]. 이러한 결과는 한국 성인의 수면 부족 문제가 심각한 수준임을 시사한다. 수면 부족 현상은 단기간의 일시적 현상이 아닌, 생활 습관 및 불규칙한 근무 형태, 디지털 기기 사용 증가 등 사회문화적 환경 변화에 따른 구조적인 문제로 해석될 수 있으며[30], 이에 따라 간호 실무에서는 개인의 수면 행태에 대한 사정뿐 아니라 지역사회 차원의 수면 건강증진 전략이 병행되어야 할 필요성이 제기된다. 특히 수면 시간은 다양한 건강지표와 밀접하게 연관되어 있다는 점에서, 성인 대상 건강증진 사업이나 만성질환 예방 교육 프로그램에서 수면 평가와 중재가 포함되어야 하며, 간호사는 수면 위생 교육, 상담 및 행동 변화 촉진을 위한 역할을 수행할 필요가 있다.

본 연구에서는 수면 부족이 스트레스 수준 증가와 밀접하게 연관되어 있음을 확인하였다. 수면 시간이 짧을수록 스트레스를 경험하는 비율이 유의하게 높았는데, 이는 2015년 우리나라 근로자 67,425명을 대상으로 실시한 지역사회건강조사(Community Health Survey) 분석 연구에서 수면 시간이 짧을수록 스트레스가 높아진다는 연구 결과와 일치하였다[31]. 또한 성인을 대상으로 한 국내 연구에서 72.5%의 대상자가 적정 수면 시간을 취하지 못하여 스트레스가 증가한다고 보고하였으며[32], 대학생을 대상으로 한 국내외 연구에서도 수면 부족이 스트레스를 증가시키고 뇌 기능과 건강에 악영향을 미친다고 보고하였다[33]. 한편, 일부 연구에서는 수면 시간이 스트레스에 영향을 주지 않는 것으로 나타났는데[34], 이는 수면과 스트레스 간의 관계가 개인과 환경의 특성 및 요인에 따라 다르게 나타날 수도 있음을 시사한다. 이처럼 수면 부족이 항상 스트레스 수준을 증가시키는 단일한 기전으로 작용하지 않으므로, 두 변수 간의 관계는 복합적이고 다양한 맥락에서 해석되어야 한다. 따라서 간호 실무에서는 대상자의 수면 상태와 스트레스 수준을 함께 사정하고, 신체적•정신적 특성을 고려한 개별적이고 통합적인 관리 방안을 모색할 필요가 있다[35].

본 연구 결과, 우리나라 성인의 고 우울 그룹은 전체의 4.2%로 매우 높은 수준임을 확인하였다. 이는 유럽 27개국에서 258,888명의 일반인을 대상으로 시행한 European Health Interview Survey에서 PHQ-8의 설문 결과 우울증 유병률이 2.1%인 것에 비해 매우 높은 수준이다[36]. 우리나라의 자살률은 OECD 국가 중 가장 높으며, 우울증 환자의 상당수가 자살 시도를 경험하는 것으로 나타났다[37]. 특히, 우울은 개인의 삶의 질을 저하시킬 뿐만 아니라 의료 이용 증가와 생산성 저하 등 사회•경제적 부담으로 이어질 수 있으므로, 국가적 차원의 우울 예방 및 중재 방안 마련을 검토할 필요가 있다.

본 연구에서 수면 부족이 우울에 미치는 영향을 확인한 결과, 수면 부족 그룹과 수면 과다 그룹은 적정 수면 그룹에 비해 우울이 발생할 위험이 각각 5.62배, 5.40배 높게 나타났다. 이는 짧은 수면 시간과 우울증 발병 위험 간의 연관성을 보고한 다수의 선행연구 결과와 일치한다[38,39]. 또한 미국 국립보건통계센터(National Center for Health Statistics)가 25,962명을 대상으로 PHQ-9과 수면 시간을 조사한 결과, 짧은 수면 시간뿐만 아니라 적정 수면 시간을 초과한 과다 수면 또한 우울증의 발병 위험을 높인다는 결과도 일치한다[40]. 그리고 성인의 수면 시간과 우울 간의 연관성을 밝히기 위한 메타분석 연구에서 짧은 수면뿐만 아니라 과다 수면도 우울 증가와 관련이 있다고 보고한 결과와도 일치한다[41]. 이러한 결과는 단순히 수면의 양이 아닌, 적절한 수면 시간의 중요성을 시사하며, 국민의 건강한 수면 습관을 위하여 수면 위생 및 적정 수면에 대한 적극적인 교육과 홍보가 필요하다. 또한 수면과 우울이 양방향으로 영향을 미칠 수 있는 복합적인 관계임을 고려하였을 때[42], 수면과 우울의 관계를 조사하는 추가적인 종단 연구가 필요할 것으로 보인다.

본 연구에서는 정신건강의 지표로 ‘정신 문제 상담 경험‘을 확인하였다. 국내 성인을 대상으로 한 연구에 따르면 우울증 환자 중 상담 경험 그룹은 35.3% 비 경험 그룹은 64.7%였으며, 상담 경험 그룹의 자살 시도 여부가 13.8%로 비 경험 그룹의 자살 시도 여부가 5.6%인 것에 비해 유의하게 높았다[43]. 이는 정신 문제 상담 경험이 중등도 이상의 정신건강 문제를 시사하는 중요한 지표로서, 정신건강 고위험군을 식별하는데 유용한 행동 기반 지표가 될 수 있음을 시사한다. 따라서 정신건강 문제 상담의 경험이 있는 대상자들에 대해서 체계적이고 지속적인 관리 방안의 모색이 필요하다.

본 연구에서는 수면 부족과 정신 문제 상담 경험이 밀접하게 연관되어 있음을 확인하였다. 수면 부족 그룹은 적정 수면 그룹보다 정신 문제 상담 경험이 있는 비율이 유의하게 높았으며, 수면 시간이 정신 문제 상담 경험에 미치는 영향을 확인한 결과 수면 부족 그룹이 적정 수면 그룹보다 정신 문제 상담을 받을 가능성이 3.57배 더 높았다. 이는 수면 부족 그룹이 적정 수면 그룹보다 정신 문제를 가지고 있을 확률과, 정신 문제의 중증도가 더 높은 것을 의미한다. 하지만 우리나라는 사회적 편견의 문제로 정신 상담을 어려워하는 경향이 있어 실제로 정신 문제를 가진 대상자는 더 많을 것으로 예상된다[44]. 수면 시간의 결정요인을 조사한 코호트 연구에 따르면, 짧은 수면은 흡연, 높은 수준의 알코올 소비, 비만, 우울증, 불안, 미혼 그리고 더 긴 근무 시간, 낮은 교육 수준과 관련이 있었다[45]. 이는 수면 문제 개선을 위해서는 개인뿐만 아니라 사회적 차원의 접근 또한 필요한 것을 시사한다. 따라서 개인에게는 건강한 수면 습관을 위한 교육 및 생활 습관 개선이 요구되며, 근무 시간 조정과 같은 적정 수면 시간 보장을 위한 정책이 필요할 것으로 보인다.

본 연구에서는 정신건강의 지표로 주관적 건강인지를 확인하였으며, 수면 시간이 주관적 건강인지에 미치는 영향을 분석한 결과, 유의한 차이는 나타나지 않았다. 이러한 결과는 수면 시간이 부족할수록 스스로 인지하는 건강 수준이 낮다는 선행연구[46]와 일관되지 않은 경향을 보였다. 이는 선행연구가 600여 명의 표본을 대상으로 한 것과 달리 본 연구는 대한민국 성인 전체를 대표하는 대규모 표본을 대상으로 실시 하였으므로, 연구 결과의 차이는 표본의 규모와 특성 차이에서 기인한 것으로 볼 수 있다. 기존의 소규모 연구들은 특정 직업군이나 연령층의 특성이 강하게 반영된 표본을 사용함으로써, 수면 시간과 주관적 건강 인지 간의 관계가 명확하게 나타났을 가능성이 있으나 본 연구는 대규모 데이터를 분석함으로써, 수면 시간이라는 변수 외에도 주관적 건강 인지를 결정하는 다양한 요인들이 복합적으로 작용했음을 알 수 있다. 따라서 수면 시간과 주관적 건강 인지 간의 관계를 매개하거나 조절하는 다양한 변인들을 탐색하는 후속 연구가 필요할 것으로 보인다.

본 연구에서는 수면 시간이 음주 빈도와 폭음 유무에 미치는 영향을 분석하였으나, 유의한 차이가 없었다. 이러한 결과는 세 나라의 대학생을 대상으로 한 연구에서 수면 시간 및 수면의 질이 음주 빈도와 유의한 관련성을 보이지 않았다는 연구 결과와는 일치하였으나[47], 수면 시간이 짧을수록 알코올 섭취량이 증가하며, 수면 부족이 음주 습관에 영향을 미친다고 보고한 선행연구[20]와는 상이한 결과를 보였다. 이러한 불일치는 수면과 음주 간의 관계가 단순한 양방향적 상관관계를 넘어, 사회심리적 요인, 개인의 대처 전략, 생활 리듬(chronotype) 등 다양한 변인의 영향을 받을 수 있기 때문으로 해석할 수 있다[48,49]. 따라서 수면과 음주 습관 간의 명확한 인과성을 규명하기 위해서는, 향후 종단 연구나 실험 연구와 같은 보다 더 정교한 연구 설계가 필요할 것으로 보인다.

본 연구는 국민건강영양조사의 대규모 데이터를 체계적으로 활용하여, 한국 성인의 수면 시간이 정신건강 및 음주 습관에 미치는 영향을 다각적으로 분석했다는 점에서 의의가 있다. 그러나 다음과 같은 한계점을 갖는다. 첫째, 본 연구는 횡단적 연구 설계로, 수면 시간과 정신건강 및 음주 습관 간의 인과 관계를 명확히 규명하는 데 한계가 있다. 둘째, 수면과 관련된 중요한 변수인 수면의 질이나 수면장애 유무를 고려하지 않고 오직 수면 시간만을 주요 변수로 분석하여, 수면의 복합적인 측면을 충분히 반영하지 못했다. 셋째, 수면 시간이 자가 보고 방식으로 측정되었기 때문에, 액티그라피와 같은 객관적인 측정 방식에 비해 회상 오류 등의 오차 발생 가능성이 크다는 한계가 있다. 따라서 향후 연구에서는 이러한 제한점을 보완하기 위해, 종단적 연구 설계를 통해 명확한 인과관계를 밝히고, 수면의 질 및 수면 장애를 포함한 다양한 수면 관련 요인을 고려하며, 객관적인 수면 측정 방법을 활용하는 노력이 필요하다.

## 결론

본 연구는 수면 부족이 만연한 현대사회에서 수면 시간이 정신건강 및 음주 습관에 미치는 영향을 확인하고자 국민건강영양조사 데이터를 활용하여 수행되었다. 연구 결과, 수면 부족 그룹은 적정 수면을 취하는 그룹에 비해 우울, 스트레스 인지, 정신 문제 상담 경험을 현저히 증가시키는 것으로 나타났다. 한편, 수면 시간은 음주 빈도 및 폭음 유무에는 유의한 영향을 미치지 않았다. 연구 결과를 바탕으로 다음과 같이 제언하고자 한다.

첫째, 본 연구는 한국 성인 중 상당수가 수면 부족 상태에 놓여 있으며, 이들이 우울과 스트레스와 같은 정신건강 문제를 더 많이 경험하고 있음을 보여준다. 따라서 정부와 공중보건 기관은 수면 부족이 정신건강에 미치는 부정적 영향에 대한 인식을 제고하고, 국민의 건강한 수면 습관 형성을 돕기 위한 정책적 노력을 기울여야 한다.

둘째, 본 연구 결과는 수면 부족이 우울 및 스트레스를 증가시키고 정신 문제 상담 경험과도 밀접하게 연관되어 있음을 보여준다. 따라서 국민의 정신건강 문제 예방 및 중재를 위한 체계적인 방안 마련이 필요하다. 특히, 정신건강 고위험군 식별에 지표가 될 수 있는 정신건강 상담에 대한 사회적 편견을 해소하고 접근성을 높이기 위해 모바일 앱이나 온라인 플랫폼을 활용한 디지털 정신건강 관리 서비스 구축과 같은 실질적인 대책 마련이 필요하다. 이를 통해 국민들이 정신 건강전문가의 도움을 더욱 쉽게 받을 수 있도록 해야 할 필요가 있다.

셋째, 본 연구에서는 수면 시간이 음주 빈도와 폭음 유무에 유의한 영향을 미치지 않는 것으로 나타났다. 그러나 수면과 음주 간의 연관성에 대한 상반된 선행 연구들이 존재하므로, 두 변수 간의 복잡한 관계와 명확한 인과 관계를 규명하기 위해 추후 종단적 연구나 더욱 정교한 설계의 연구가 수행되어야 할 것이다.

## Figures and Tables

**Table 1.** General Characteristics, Sleep Duration, Mental Health, and Alcohol Use of the Participants (N = 1,656)

Variables	Categories	n	%
Sex	Men	905	60.2
	Women	751	39.8
Age (years)	19~29	363	25.3
	30~39	423	26.4
	40~49	401	23.9
	50~59	351	19.4
	60~64	118	5.0
Education	≤ Elementary school	48	2.4
	Middle school	65	3.1
	High school	585	36.3
	≥ College or university	958	58.2
Income status	Low	302	18.6
	Low to moderate	385	24.5
	Moderate to high	474	29.1
	High	495	27.8
Marital status	Married	1,142	64.6
	Others	514	35.4
Regular worker	Yes	932	56.8
	No	724	43.2
Sleep duration (hours)	≤ 6	669	41.5
	7~9	966	57.1
	≥ 10	21	1.4
Alcohol drinking	Low-risk drinking	1,160	69.4
	High-risk drinking	496	30.6
Binge drinking (per drinking occasion)	Yes	682	43.3
	No	974	56.7
Stress	Low	1,186	70.2
	High	470	29.8
Depression	Low	1,591	95.8
	High	65	4.2
Perceived health status	Good	1,226	73.7
	Bad	430	26.3
Psychiatrist visit (in past 1 year)	Yes	48	2.8
	No	1,608	97.2

**Table 2.** Comparison of Mental Health and Alcohol Use Across Sleep Duration Groups (N = 1,656)

Variables	Categories	Sleep duration (hours)			Rao-scott χ^2^	*p*
		≤ 6	7~9	≥ 10		
Alcohol drinking	Low-risk drinking	462 (68.3)	682 (69.9)	16 (75.9)	1.00	.721
	High-risk drinking	207 (31.7)	284 (30.1)	5 (24.1)		
Binge drinking (per drinking occasion)	Yes	279 (43.6)	393 (42.7)	10 (59.7)	2.80	.430
	No	390 (56.4)	573 (57.3)	11 (40.3)		
Stress	Low	443 (64.6)	727 (74.1)	16 (80.6)	18.28	< .001
	High	226 (35.4)	239 (25.9)	5 (19.4)		
Depression	Low	625 (92.4)	947 (98.4)	19 (90.4)	37.20	< .001
	High	44 (7.6)	19 (1.6)	2 (9.6)		
Perceived health status	Good	467 (70.9)	745 (75.7)	14 (74)	4.84	.126
	Bad	202 (29.1)	221 (24.3)	7 (26)		
Psychiatrist visit (in past 1 year)	Yes	31 (4.7)	16 (1.5)	1 (2.8)	15.14	< .001
	No	638 (95.3)	950 (98.5)	20 (97.2)		

Values are presented as n (%).

**Table 3.** The Impact of Sleep Duration on Alcohol Use, Adjusted for General Characteristics (N = 1,656)

Variables	Categories	Alcohol drinking			Binge drinking		
		OR	95% CI	*p*	OR	95% CI	*p*
Sex^†^ (ref= Women)	Men	2.39	1.84~3.10	< .001	1.80	1.39~2.34	< .001
Age (years)^†^ (ref= 60~64)	19~29	0.93	0.48~1.79	.827	3.54	1.83~6.85	< .001
	30~39	1.30	0.76~2.25	.340	2.82	1.58~5.05	< .001
	40~49	1.28	0.76~2.17	.350	2.35	1.32~4.21	.004
	50~59	1.27	0.75~2.17	.372	1.57	0.89~2.75	.116
Education^†^ (ref= ≥ College or university)	≤ Elementary school	1.63	0.74~3.56	.222	1.65	0.76~3.57	.205
	Middle school	1.15	0.62~2.13	.656	1.63	0.89~2.99	.116
	High school	0.92	0.70~1.22	.570	1.22	0.93~1.61	.148
Income status^†^ (ref= High)	Low	1.18	0.78~1.78	.441	1.54	1.05~2.27	.028
	Low to moderate	1.33	0.93~1.89	.114	1.46	1.04~2.05	.029
	Moderate to high	1.36	0.96~1.92	.081	1.33	0.97~1.83	.073
Marital status^†^ (ref= Others)	Married	1.44	0.96~2.15	.077	0.75	0.53~1.06	.100
Regular worker^†^ (ref= No)	Yes	1.04	0.79~1.38	.765	1.09	0.83~1.43	.518
Sleep duration (hours) (ref= 7~9)	≤ 6	0.99	0.79~1.24	.914	1.06	0.82~1.37	.635
	≥ 10	0.86	0.21~3.54	.837	1.35	0.47~3.87	.572

OR = Odds ratio; CI = Confidence interval; ref = Reference.

† Adjustment variables: Sex, age, education, income status, marital status, regular worker.

**Table 4.** Associations between Sleep Duration and Mental Health Outcomes, Adjusted for General Characteristic (N = 1,656)

Variables	Categories	Stress			Depression			Perceived health status			Psychiatrist visit		
		OR	95% CI	*p*	OR	95% CI	*p*	OR	95% CI	*p*	OR	95% CI	*p*
Sex^†^ (ref= Women)	Men	0.89	0.70~1.12	.327	0.82	0.46~1.47	.510	0.78	0.60~1.02	.066	0.52	0.27~0.99	.049
Age (years)^†^ (ref= 60~64)	19~29	7.27	3.29~16.04	< .001	3.75	0.62~22.65	.149	0.51	0.26~1.00	.049	0.89	0.10~8.30	.920
	30~39	7.66	3.54~16.59	< .001	3.16	0.64~15.49	.157	0.86	0.47~1.61	.646	0.41	0.04~4.04	.444
	40~49	5.36	2.47~11.62	< .001	1.82	0.33~9.90	.489	1.25	0.71~2.23	.439	1.23	0.16~9.49	.844
	50~59	2.81	1.40~5.62	.004	1.05	0.24~4.59	.945	1.30	0.76~2.21	.335	1.83	0.28~12.17	.530
Education^†^ (ref= ≥ College or university)	≤ Elementary school	3.95	1.76~8.89	< .001	2.85	0.70~11.59	.143	2.86	1.42~5.79	.003	0.82	0.09~7.37	.858
	Middle school	2.07	1.01~4.21	.046	2.92	0.98~8.66	.054	2.28	1.17~4.42	.015	1.64	0.36~7.51	.526
	High school	0.94	0.70~1.26	.688	0.92	0.50~1.68	.776	1.18	0.89~1.56	.259	0.90	0.43~1.86	.777
Income status^†^ (ref= High)	Low	1.99	1.34~2.97	< .001	2.48	1.02~6.05	.045	2.20	1.46~3.31	<.001	2.22	0.92~5.40	.078
	Low to moderate	1.64	1.14~2.35	.007	2.63	1.05~6.56	.039	1.63	1.12~2.38	.011	1.46	0.53~4.01	.463
	Moderate to high	1.29	0.91~1.84	.157	1.15	0.44~2.98	.777	1.36	0.94~1.95	.099	1.35	0.54~3.36	.519
Marital status^†^ (ref= Others)	Married	1.22	0.84~1.76	.298	2.53	1.06~6.05	.037	0.53	0.35~0.79	.002	0.35	0.13~0.96	.042
Regular worker^†^ (ref= No)	Yes	1.12	0.84~1.48	.446	0.64	0.34~1.22	.172	0.87	0.65~1.18	.375	1.15	0.58~2.28	.687
Sleep duration (hours) (ref= 7~9)	≤ 6	1.65	1.29~2.12	< .001	5.62	3.00~10.52	< .001	1.30	0.99~1.71	.058	3.57	1.82~7.01	< .001
	≥ 10	0.57	0.23~1.39	.215	5.40	1.77~16.47	.003	1.01	0.43~2.41	.973	1.31	0.15~11.30	.807

OR = Odds ratio; CI = Confidence interval; ref = Reference.

† Adjustment variables: Sex, age, education, income status, marital status, regular worker.

## Data Availability

This study used secondary data from the Korea National Health and Nutrition Examination Survey (KNHANES), which is publicly available at https://knhanes.kdca.go.kr. The authors had no access to identifying participant information.
